# Assessing the Impact of a Serious Game (MedSMARxT: Adventures in PharmaCity) in Improving Opioid Safety Awareness Among Adolescents and Parents: Quantitative Study

**DOI:** 10.2196/51812

**Published:** 2023-12-07

**Authors:** Olufunmilola Abraham, Tyler James McCarthy, Jen Zaborek

**Affiliations:** 1 Social and Administrative Sciences Division School of Pharmacy University of Wisconsin-Madison Madison, WI United States; 2 Biostatistics and Medical Informatics School of Medicine and Public Health University of Wisconsin-Madison Madison, WI United States

**Keywords:** adolescent, opioid, medication safety, serious games, gamification, health behavior

## Abstract

**Background:**

The opioid crisis continues to worsen across the United States, affecting people of all demographics. Few evidence-based interventions exist for educating families, particularly those with adolescents, about opioid prescription safety. Serious games have demonstrated impacts in improving medication-related outcomes for various health conditions. The characterizing goal of this serious game is to improve opioid safety knowledge and awareness among adolescents and their families.

**Objective:**

This study evaluated the impact of a serious game, MedSMAR_x_T: Adventures in PharmaCity, designed to foster opioid safety awareness among adolescents and their parents.

**Methods:**

A national sample of parents and their adolescent children was recruited through Qualtrics research panels, social media, listservs, and snowball sampling. Eligible participants were adolescents aged between 12 and 18 years and their parents. Study participants were required to reside in the United States; speak, read, and understand English; and have access to a computer with a webcam. Parent-child dyads completed pregame and postgame surveys and participated in gameplay for up to 30 minutes. Primary outcome scales have been previously evaluated by the study team.

**Results:**

A total of 60 adolescent participants and 68 parent participants met full attention criteria for inclusion in this study. Statistical analysis confirmed that both adolescents’ and parents’ concept scores improved from baseline regarding opioid safety self-efficacy (adolescent: mean 0.35, SD 0.60; *P*<.001; parent: mean 0.28, SD 0.42; *P*<.001), perceived knowledge (adolescent: mean 1.08, SD 1.04; *P*<.001; parent: mean 0.56, SD 0.55; *P*<.001), behavioral intent (adolescent: mean 0.26, SD 0.39; *P*<.001; parent: mean 0.25, SD 0.32; *P*<.001), safe storage (adolescent: mean 0.12, SD 0.27; *P*<.001; parent: mean 0.03, SD 0.11; *P*=.03), disposal knowledge (adolescent: mean 0.10, SD 0.27; *P*=.006; parent: mean 0.07, SD 0.16; *P*<.001), and knowledge about misuse behavior (adolescent: mean 0.05, SD 0.14; *P*=.002; parent: mean 0.04, SD 0.10; *P*<.001). Participant groups, stratified by who completed and who did not complete gameplay, improved their knowledge and awareness, with no significant differences between subgroups.

**Conclusions:**

The use of this serious game to improve opioid prescription safety practices among parents and adolescents was supported by the study findings. MedSMAR_x_T: Adventures in PharmaCity is an intervention with the capability of teaching parents and adolescents about safe opioid prescription practices. Further studies and game refinement are needed to demonstrate the effectiveness of a game-based intervention in clinical settings and community pharmacies.

## Introduction

### Background

The opioid epidemic has had a devastating impact on the US population in recent years. Although opioid medication presents dangers in terms of side effects and addiction potential, they are still a necessary part of treatment for many. The potential dangers of these medications only increase when they are prescribed to adolescents or their parents. Having these medications at home presents the risk of opioid misuse. Misuse can be defined as taking a medication in any way other than how it was prescribed. This includes taking a higher dosage or more frequently than prescribed, taking medication for euphoric effects, or taking a medication not prescribed to oneself. In 2019, overall, 7.6% of US adolescents reported misuse, related primarily to prescription opioids [1]. Adolescents are most likely to be prescribed hydrocodone, oxycodone, and codeine even while the black box warning for codeine denotes high risk for complications [2].

Moreover, opioid death rates in adolescents have increased by >300% from 1999 to 2021 [3,4]. In summary, opioid misuse is a growing cause of death among adolescents, in part owing to high rates of prescription misuse. Although many adolescents are prescribed opioids every year, 64% of clinicians do not have a standardized protocol for prescribing pain management [5]. The lack of protocol and assumptions that medications that work for adults also work for children, compounds with additional factors such as socioeconomic status, sex, employment, and others. Therefore, there is a certain need for tailored education for adolescents regarding opioid safety [6].

Given these trends, prevention of opioid misuse is critical in practitioners’ attempts to curb the opioid epidemic. The Pediatric Pharmacy Advocacy Group recommends educating children and their families about proper administration, storage, and disposal by empowering parents to talk with their children about prescription opioid misuse [7]. A family-driven intervention is essential, given the known misuse behaviors that occur within households. Parents often model inappropriate prescription opioid use by sharing unused medications with their children to treat minor injuries; teens take medications from home medicine cabinets; or parents give children incorrect dosages [8-10]. Adults also report an intention to keep leftover opioid medications for later use [11]. This inappropriate use of prescriptions supports the need for expert guidance for families to discuss prescription opioid safety at home.

Teens report parents as useful resources for information about opioid safety, especially mothers [7,12]. Specifically, this study with adolescents indicated major themes of educational interest related to perceptions about opioids and misuse, prevalence of prescription opioid misuse, reasons for misuse, consequences of misuse, and common information sources [12]. These themes supported the use of various scales to evaluate adolescent awareness based on interest and knowledge gaps.

A clear opportunity exists to positively influence family communication and practices around opioid safety and responsible management of prescription [8,13]. Currently, there are no standard ways to encourage parents to have conversations about medication safety with their children. Many of the available education programs regarding medication management and safety are targeted toward schools (eg, Rx for Addiction and Medication Safety) [14]. The available patient medication safety plans are not focused on opioid management and lack scientifically based foundations.

Previous studies found that adolescents aged between 12 and 18 years and parents desired innovative technologies [15-19]. In collaboration with community partners and game developers, the study team created an innovative, novel, and engaging game. The serious game, MedSMAR_x_T: Adventures in PharmaCity, was created to educate adolescents and their families about safe opioid use and was developed using a serious game behavior change framework for improving medication safety in community settings [20]. This framework is based on existing psychosocial behavioral interventions and theories intended to positively influence behavior and cognition for health-related conditions and mental and physical well-being [20]. Additional previous studies have evaluated the game’s educational content and impressions of pharmacists. Pharmacists reported that the educational content of the game was accurate and that the game would be valuable to pharmacists, parents, and teens [21].

Serious games have demonstrated not only acceptability but also robust influence on relevant outcomes. Previous studies have shown that video games often lead to great engagement with educational material, which can facilitate the retention of pertinent knowledge [22,23]. Studies have shown video games to be useful for adolescents and adults to learn more about various health-related topics. Serious games have shown the ability to produce the desired results in health-related contexts such as adolescent and young adult patients’ cancer treatment adherence, cancer-related self-efficacy, and knowledge. Other serious games were developed to facilitate medication adherence in patients with HIV, formation of healthy eating habits in children with Celiac disease, and amelioration of attention-deficit/hyperactivity disorder symptoms and their ongoing assessment [24-31]. MedSMAR_x_T: Adventures in PharmaCity has been previously evaluated by the study team with a national sample of youth. This study found that adolescents improved on most of the 10 key concepts related to opioid safety [32]. Early assessment presented in the study necessitated further replication of the game’s effects and an evaluation of parent concept score outcomes. However, further study is needed to understand the effects of the intervention on relevant scales to identify and develop valid benchmarks for opioid safety awareness among adolescents.

### Study Objective

This study aimed to evaluate the impact of MedSMAR_x_T: Adventures in PharmaCity on an opioid safety awareness scale among adolescents and the adolescents’ parent as dyads. The primary outcome was a change in concept scores for the 10 concepts implicated in opioid medication safety used in previous studies by the study team. The secondary outcome was to test whether differences in family communication patterns correlate with changes in concept scores.

## Methods

### Ethical Considerations

This study was approved by the University of Wisconsin-Madison institutional review board (2020-1638). Participants were compensated US $30 via Amazon e–gift card for their time, given to the parent participant.

### Recruitment

Parent and adolescent dyads were recruited from April 2021 to October 2021 using convenience sampling through existing Qualtrics research panels, social media (Facebook, Twitter, Reddit, and Instagram), email listservs (university’s mass email system and laboratory-specific listservs), and snowball sampling. The sampling framework included adolescents and parents from across the nation who met eligibility criteria. Interested participants were recruited with the understanding that they would play an educational game about opioid safety. To be eligible for this study, adults had to be parents of adolescents aged between 12 and 18 years, adolescents had to be aged between 12 and 18 years, and both parents and youth had to be able to communicate with and comprehend English and have access to a computer or laptop with functional webcam. Parents indicated interest via a Qualtrics survey. Following survey submission, parents were contacted within a week to schedule their study session and received 3 weekly reminders if needed. Parental consent and child assent were obtained before initiation of the study session. All study sessions were conducted via the Webex web-based platform.

### Data Collection

Data were collected through pregame surveys, 30 minutes of gameplay, and postgame surveys. Pregame and postgame opioid-related survey questions were adapted from various validated surveys and scales or created by the investigators [33,34]. Questions from a Wisconsin state-wide survey assessed participants’ knowledge about safe prescription opioid storage and disposal [12,35]. Questions measuring self-efficacy of prescription opioid use and other learning objectives were adapted from the Medication Understanding and Use Self-Efficacy (MUSE) scale and a survey assessing workplace safety and health knowledge among adolescents [36,37]. Questions measuring participants’ knowledge about naloxone (Narcan) were adapted from the Maryland Opioid Survey Summary Report [38]. The Revised Family Communication Patterns (RFCP) instrument was used to measure 2 underlying dimensions of family communication patterns: conversation orientation (15 items) and conformity orientation (11 items) [39]. Parent and child participants were presented with their respective versions of the RFCP. All scales, excluding RFCP, had been previously used by the study team [12,33,34]. All surveys were hosted through the web-based Qualtrics platform.

### Pregame Survey

The pregame survey consisted of 87 items. In total, 59 items were used to measure participants’ baseline opioid knowledge; knowledge about safety, disposal, and misuse; and perceived effect that a serious game may have on opioid safety awareness. These concepts were measured using “yes,” “no,” and “don’t know” answers or a 5-point Likert scale. Overall, 2 items were used as attention check questions to prevent straight-lining by asking participants to select a specific option within the 5-point Likert scale. Attention check questions ask the participant to answer a question in a specific way to demonstrate that they are reading each question, rather than randomly selecting responses or providing the same responses for all questions. In total, 26 items were from the RFCP instrument. In addition to the 87 items, 11 demographic questions asked for parent participants’ characteristics, including age, sex, race, ethnicity, income, employment status, languages spoken, number of children at home, marital status, and highest level of education of the participant and their partner. Then, 7 demographic questions asked for child participants’ characteristics, including age, sex, race, ethnicity, languages spoken, number of children in the household, and what grade they were in for the 2020 to 2021 school year. The pregame survey questions are included in Multimedia Appendix 1.

### The MedSMARxT: Adventures in PharmaCity Game

The serious game, MedSMAR_x_T: Adventures in PharmaCity, was developed and tailored to educate adolescents and their families about safe opioid use, storage, and disposal [20]. It is played via the web browser on a computer. The game is organized as a responsive narrative where players must make safe choices to progress through the levels. MedSMAR_x_T: Adventures in PharmaCity consists of 5 levels and provides immediate feedback to the player if they made a wrong decision. Participants play as “Shan,” an anthropomorphized sheep. The player’s goal is to guide Shan to make the right choices for opioid use and maintenance. Level 1, A Quiet Sunday Afternoon, is focused on teaching the player about safe storage of opioids. Level 2, Monday Morning Bus Ride, adds a real-life scenario to the game where the player is in pain and forgot that there was an important assignment due that day. Level 3, A Persuasive Speech at School, focuses on teaching the player to not take others’ pain medication and the negative consequences of taking someone else’s prescription medication. Level 4, Bus Ride Home, teaches the player to not share their medication with others and the negative consequences of sharing medications with others and introduces the use of Narcan. Level 5, Last Minute Chore, shows the player the correct way to dispose of an opioid medication.

### Postgame Survey

The postgame survey consisted of 104 items. The same 59-item, baseline, opioid-related questions in the pregame survey were used along with an additional 40 questions asking about participants’ perspectives regarding the MedSMAR_x_T: Adventures in PharmaCity game, 3 questions asking about previous opioid experience, and 2 attention check questions. This survey included 5-point Likert scale questions; multiple choice questions; free-response questions; and questions with “yes,” “no,” and “unsure” as answers. The postgame survey is included in Multimedia Appendix 2.

### Procedure

Each parent-child dyad completed the data collection session independently, in a private location of their choice, to ensure that their answers were not influenced by the presence of the parent or child. Before each session, participants were screened again for eligibility by the research staff. Once eligibility was confirmed for a second time, participants were asked to complete the pregame survey, play the MedSMAR_x_T: Adventures in PharmaCity game for up to 30 minutes, and complete the postgame survey. Gameplay sessions were recorded for observation. The researcher who introduced the game to the participants was a research staff member.

### Statistical Analysis

Survey questions were divided into 10 categories to represent key concepts and summarized into concept scores. Pregame and postgame surveys had concept scores that ranged from 1 to 5 when the items were Likert scales and from 0% to 100% when the items were knowledge scores; changes in concept scores, from pregame survey to postgame survey, were calculated for each participant and were the primary intended outcomes.

Concept score changes were described using overall mean (SD) and were stratified by sex, race, school, grade, and age. Differences in concept score changes were analyzed using the Kruskal-Wallis tests for categorical characteristics, correlation tests for continuous characteristics, and Jonckheere-Terpstra tests for categorical characteristics with a natural ordering (parents’ education and income). When a categorical characteristic was found to be significant, pairwise testing was conducted using Mann-Whitney-Wilcoxon tests.

Primary analysis included participants who accessed the game based on IP (located within the United States) and satisfied the attention checks for both pregame and postgame surveys. Thus, not all child-parent dyads met the full attention criteria; participants who failed the attention check were not included in the analysis. Secondary analyses aimed to (1) assess the differences between participants included in primary analysis and those who did not meet the full attention criteria and (2) assess the associations of the levels played and the length of play with concept scores. The participants who did not meet the full attention criteria either did not access the game or did not self-report playing the game but met other attention criteria (ie, select “a great deal” when asked). No *P* value adjustments were made to account for inflated type-1 error rate. Significance was assessed with a type 1 error rate of α=.05 level. All statistical analyses were performed using R statistical software (version 4.1.3; R Foundation for Statistical Computing).

## Results

### Results From Child Participants

There were 60 child participants who met the full attention criteria on pregame and postgame surveys and accessed the game. Among these participants, 53% (32/60) identified as male, 60% (36/60) identified as White, and the mean age was 14.02 (SD 1.59) years. Child demographics are reported in Table 1.

Child participants significantly improved on all concept scores except for Narcan knowledge (mean change 0.05, SD 0.36; *P*=.59), misuse harm (mean change −0.06, SD 0.65; *P*=.77), and self-efficacy–MUSE (mean change 0.07, SD 0.82; *P*=.52; Table 2). Child results stratified by demography are reported in Table S1 in Multimedia Appendix 3.

Although all participants (60/60, 100%) accessed and played the game, in-game data were not available for all participants owing to browser and internet access differences. Of the 97% (58/60) child participants with in-game data, 79% (46/58) played all 5 levels. The participants who partially completed the game played an average of 3.18 (SD 0.40) levels (Table 3). There were no differences in concept scores between completers and noncompleters. Completers had a short length of play (completers: mean 18.66, SD 3.44 minutes vs noncompleters: mean 23.29, SD 1.41 minutes; *P*<.001).

The number of in-game opioid successes was not associated with children’s RFCP conformity (ρ=–0.23, 95% CI –0.46 to 0.04; *P*=.09) or conversation scores (ρ=0.08, 95% CI –0.19 to 0.33; *P*=.58). Opioid knowledge improvement was inversely associated with RFCP conversation (ρ=–0.28, 95% CI –0.50 to –0.03; *P*=.03; Table S2 in Multimedia Appendix 3).

**Table 1:** Child participants’ demographic characteristics (n=60).

Characteristics		Participants
**Grade, n (%)**		
	5	1 (2)
	6	6 (10)
	7	15 (25)
	8	11 (18)
	9	10 (17)
	10	9 (15)
	11	5 (8)
	12	3 (5)
Age (y), mean (SD)		14.02 (1.59)
**Sex^a^, n (%)**		
	Female	21 (35)
	Male	32 (53)
	Other: please specify	7 (12)
**Race or ethnicity^b^, n (%)**		
	White or Caucasian	36 (60)
	Black or African American	9 (15)
	Hispanic or Latinx	3 (5)
	Asian	0 (0)
	American Indian or Alaska Native	1 (2)
	Native Hawaiian or other Pacific Islander	0 (0)
	>1 selected	8 (13)
	Other: please specify	3 (5)

^a^In total, 3 options were presented to the participants to select for their sex: “Male,” “Female,” and “Other: Please specify.”

^b^If participants only selected 1 category, that was their defined race; all other combinations of selections were defined as “Other.”

**Table 2:** Concept scores among child participants. Italicized values are statistically significant changes.

Characteristics		Self-efficacy–MUSE^a^	Self-efficacy–opioid safety	Perceived knowledge	Misuse harm	Behavioral intent	Safe storage	Safe disposal	Opioid knowledge	Narcan knowledge	Misuse behavior
Pregame Cronbach α		.85	.73	.90	.89	.64	.78	.86	.63	.76	.70
**Overall**											
	Participants, (n=60), n (%)	60 (100)	60 (100)	59 (98)	59 (98)	58 (97)	60 (100)	60 (100)	60 (100)	13 (22)	60 (100)
	Score, mean (SD)	0.07 (0.82)	*0.35 (0.60)*	*1.08 (1.04)*	−0.06 (0.65)	*0.26 (0.39)*	*0.12 (0.26)*	*0.10 (0.27)*	*0.08 (0.20)*	0.05 (0.36)	*0.05 (0.14)*
	Kruskal-Wallis *P* value	.52	*<.001*	*<.001*	.77	*<.001*	*<.001*	*.006*	*<.001*	.59	*.002*

^a^MUSE: Medication Understanding and Use Self-Efficacy.

**Table 3:** Examining the differences between child participants who completed all 5 levels of the game and those who did not.

Variables			Noncompleters (n=11)		Completers (played all 5 levels; n=46)		*P* value	
Behavioral intent, mean (SD)			0.22 (0.29)		0.27 (0.41)		.45	
Misuse behavior, mean (SD)			−0.02 (0.09)		0.07 (0.16)		.06	
Misuse behavior 2, mean (SD)^a^			0 (0)		0.04 (0.29)		.62	
Misuse harm, mean (SD)			0.11 (0.19)		−0.01 (0.43)		.22	
Narcan knowledge, mean (SD)			0 (0)		0.07 (0.41)		.78	
Opioid knowledge—harming teens, mean (SD)^b^			0.27 (0.47)		0.15 (0.36)		.35	
Opioid knowledge, mean (SD)			0.05 (0.16)		0.08 (0.21)		.62	
Perceived knowledge, mean (SD)			1.39 (0.78)		1.13 (0.82)		.23	
Safe disposal, mean (SD)			0.02 (0.09)		0.12 (0.31)		.33	
Safe storage, mean (SD)			0.16 (0.30)		0.12 (0.26)		.52	
Self-efficacy—opioid safety, mean (SD)			0.40 (0.48)		0.38 (0.54)		.98	
Self-efficacy–MUSE^c^, mean (SD)			0.27 (0.63)		0.10 (0.78)		.46	
Length of play, mean (SD)			23.29 (1.41)		18.66 (3.44)		<.001	
Number of levels played, mean (SD)			3.18 (0.40)		N/A^d^		N/A	
**Sex, n (%)**								.86
	Female	3 (27)		16 (35)				
	Male	7 (64)		25 (54)				
	Other: please specify	1 (9)		5 (11)				
Age (y), mean (SD)			13.45 (1.44)		14.09 (1.52)		.22	
**Race (grouping 1), n (%)**								.93
	A—White or Caucasian	6 (55)		29 (63)				
	B—Black or African American	2 (18)		6 (13)				
	C—Hispanic or Latinx	2 (18)		6 (13)				
	D—Other or missing	1 (9)		5 (11)				
Race (grouping 2; B—non-White), n (%)^e^			5 (45)		17 (37)		.60	

^a^Individual question asking, “Is it okay to take someone else’s opioid medication if you have had the same prescription in the past?”

^b^Individual question asking, “Is the opioid crisis harming teenagers in the United States?”

^c^MUSE: Medication Understanding and Use Self-Efficacy.

^d^N/A: not applicable.

^e^Race grouping 2 considers participants who selected “White” as 1 category and every other option as “non-White.”

### Results From Parent Participants

There were 68 parent participants who met the full attention criteria on pregame and postgame surveys and accessed the game. Among these participants, 93% (63/68) identified as female, 78% (53/68) identified as White, and the mean age was 45.69 (SD 5.33) years. Parent demographics are reported in Table 4.

Parent participants significantly improved on all concept scores except for Narcan knowledge (mean change 0, SD 0.20; *P*=.87), opioid knowledge (mean change 0.01, SD 0.08; *P*=.33), and self-efficacy–MUSE (mean change 0.09, SD 0.54; *P*=.21; Table 5). Parent results stratified by demographic variables are reported in Table S3 in Multimedia Appendix 3.

Of the 99% (67/68) of parent participants with in-game data, 61% (41/67) played all 5 levels. The participants who partially completed the game played an average of 3.35 (SD 0.49) levels (Table 6). Completers had a short length of play (completers: mean 21.55, SD 5 min vs noncompleters: mean 23.34, SD 2.10 min; *P*=.007) and great improvement in safe disposal knowledge (completers: mean 0.10, SD 0.19 min vs noncompleters: mean 0.01, SD 0.03 min; *P*=.009).

The number of in-game opioid successes was not associated with parents’ RFCP conformity (ρ=–0.14, 95% CI –0.37 to 0.10; *P*=.25) or conversation scores (ρ=–0.07, 95% CI –0.31 to 0.17; *P*=.56). RFCP conversation was positively associated with baseline opioid knowledge (ρ=0.28, 95% CI 0.04-0.48; *P*=.02) and self-efficacy–MUSE (ρ=0.26, 95% CI 0.02-0.47; *P*=.03; Table S4 in Multimedia Appendix 3).

**Table 4:** Parent participants’ demographic characteristics (n=68).

Characteristics			Participants
**Level of education, n (%)**			
	High school	3 (4)	
	Associates or trade school	8 (12)	
	Bachelor’s degree	26 (38)	
	Master’s degree or PhD	28 (41)	
Age (y), mean (SD)			45.69 (5.33)
**Sex^a^, n (%)**			
	Female	63 (93)	
	Male	5 (7)	
	Other: please specify	0 (0)	
**Race or ethnicity^b^, n (%)**			
	White or Caucasian	53 (78)	
	Black or African American	6 (9)	
	Hispanic or Latinx	3 (4)	
	Asian	2 (3)	
	American Indian or Alaska Native	0 (0)	
	Native Hawaiian or other Pacific Islander	0 (0)	
	>1 selected	4 (6)	
	Other: please specify	0 (0)	
**Income (US $), n (%)**			
	<25,000	2 (3)	
	25,001-50,000	8 (12)	
	50,001-100,000	23 (34)	
	100,001-250,000	33 (49)	
	250,001-500,000	2 (3)	
**Employment status, n (%)**			
	Employed part time, unemployed, retired, and other	35 (51)	
	Employed full time	33 (49)	
**RFCP^c^, mean (SD)**			
	Conversation	4.25 (0.40)	
	Conformity	2.61 (0.45)	

^a^In total, 3 options were presented to the participants to select for their sex: “Male,” “Female,” and “Other.”

^b^If participants only selected 1 category, that was their defined race; all other combinations of selections were defined as “Other.”

^c^RFCP: Revised Family Communication Patterns.

**Table 5:** Concept scores among parent participants. Italicized values represent statistically significant differences.

Characteristics		Self-efficacy–MUSE^a^	Self-efficacy–opioid safety	Perceived knowledge	Misuse harm	Behavioral intent	Safe storage	Safe disposal	Opioid knowledge	Narcan knowledge	Misuse behavior
Pregame Cronbach α		.81	.74	.93	.94	.48	.51	.57	.41	.52	.41
**Overall**											
	Participants (n=68), n (%)	68 (100)	*68 (100)*	*68 (100)*	*68 (100)*	*68 (100)*	*68 (100)*	*68 (100)*	68 (100)	57 (84)	*68 (100)*
	Score, mean (SD)	0.09 (0.54)	*0.28 (0.42)*	*0.56 (0.55)*	*0.10 (0.41)*	*0.25 (0.32)*	*0.03 (0.11)*	*0.07 (0.16)*	0.01 (0.08)	0 (0.20)	*0.04 (0.10)*
	Kruskal-Wallis *P* value	.21	*<.001*	*<.001*	*.04*	*<.001*	*.02*	*<.001*	.33	.87	*<.001*

^a^MUSE: Medication Understanding and Use Self-Efficacy.

**Table 6:** Examining the differences between parent participants who completed all 5 levels of the game and those who did not.

Variables		Noncompleters (n=26)	Completers (played all 5 levels; n=41)	*P* value	
Behavioral intent, mean (SD)		0.21 (0.28)	0.29 (0.34)	.23	
Misuse behavior, mean (SD)		0.03 (0.09)	0.06 (0.11)	.20	
Misuse behavior 2, mean (SD)^a^		0 (0)	0.02 (0.16)	.43	
Misuse harm, mean (SD)		0.05 (0.34)	0.13 (0.46)	.49	
Narcan knowledge, mean (SD)		0.03 (0.22)	−0.02 (0.19)	.22	
Opioid knowledge—harming teens, mean (SD)^b^		0.04 (0.20)	0 (0.22)	.47	
Opioid knowledge, mean (SD)		0 (0.06)	0.01 (0.08)	.33	
Perceived knowledge, mean (SD)		0.43 (0.52)	0.65 (0.57)	.12	
Safe disposal, mean (SD)		0.01 (0.03)	0.10 (0.19)	.009	
Safe storage, mean (SD)		0.02 (0.10)	0.04 (0.12)	.39	
Self-efficacy—opioid safety, mean (SD)		0.24 (0.37)	0.30 (0.46)	.55	
Self-efficacy–MUSE^c^, mean (SD)		0.08 (0.59)	0.10 (0.52)	.72	
Length of play, mean (SD)		23.34 (2.10)	21.55 (5)	.007	
Number of levels played, mean (SD)		3.35 (0.49)	N/A^d^	N/A	
**Sex, n (%)**					.06
	Female	26 (100)	36 (88)		
	Male	0 (0)	5 (12)		
	Other: please specify	0 (0)	0 (0)		
Age (y), mean (SD)		46.77 (5.10)	44.95 (5.47)	.27	
**Race (grouping 1), n (%)**					.16
	A—White or Caucasian	22 (85)	30 (73)		
	B—Black or African American	3 (12)	3 (7)		
	C—Hispanic or Latinx	0 (0)	7 (17)		
	D—Other or missing	1 (4)	1 (2)		
Race (grouping 2; B—non-White), n (%)^e^		4 (15)	11 (27)	.27	
**Income (US $), n (%)**					.38^f^
	<25,000	1 (4)	1 (2)		
	25,001-50,000	5 (19)	3 (7)		
	50,001-100,000	8 (31)	15 (37)		
	100,001-250,000	11 (42)	21 (51)		
	250,001-500,000	1 (4)	1 (2)		
**Education, n (%)**					.27^f^
	High school	0 (0)	3 (7)		
	Associates or trade school	4 (15)	4 (10)		
	Bachelor’s degree	14 (54)	11 (27)		
	Master’s degree or PhD	8 (31)	20 (49)		

^a^Individual question asking, “Is it okay to take someone else’s opioid medication if you have had the same prescription in the past?”

^b^Individual question asking, “Is the opioid crisis harming teenagers in the United States?”

^c^MUSE: Medication Understanding and Use Self-Efficacy.

^d^N/A: not applicable.

^e^Race grouping 2 considers participants who selected “White” as a category and every other option as “non-White.”

^f^Calculated from Mann-Whitney-Wilcoxon test.

### Knowledge and Self-Efficacy Within Families

Baseline scores for opioid knowledge (ρ=0.04, 95% CI –0.22 to 0.30; *P*=.75), self-efficacy–learning objectives (ρ=0.12, 95% CI –0.14 to 0.37; *P*=.36), and self-efficacy–MUSE (ρ=0, 95% CI –0.26 to 0.26; *P*=.99) were not related within parent-child pairs. No other concept scores were tested for parent-child associations.

## Discussion

### Principal Findings

The results of this study demonstrate that, for both adolescents and parents, the MedSMAR_x_T: Adventures in PharmaCity game increased the concept scores on opioid safety self-efficacy, perceived knowledge, behavioral intent, safe storage and disposal knowledge, and knowledge about misuse behavior. The results support the potential efficacy of the intervention in educating adolescents and their families and supporting healthy behaviors with prescription opioids. In our previous study that evaluated the Adolescent Opioid Safety and Learning scale–related outcomes of MedSMAR_x_T: Adventures in PharmaCity, we found that participants (adolescents) improved in all concept scores, except for Narcan knowledge and safe storage [32-34]. Child Narcan knowledge scores exhibited great reduction in response, because if participants selected that they had not heard of Narcan, the Narcan knowledge questions were skipped. Therefore, several adolescents in the study did not learn about Narcan. This is in part owing to the inherent design of the game. Specifically, in level 4, if the player does not make the incorrect choice of offering a stranger in pain a pill, they would not experience the scene involving Narcan. When taken in conjunction with this study, the cumulative results suggest that the desired outcome of increasing opioid safety awareness and self-efficacy, particularly in adolescents, is possible. The suboptimal Cronbach α scores indicate that future studies are needed to further refine a scale related to opioid awareness in youth.

Although opioid safety self-efficacy significantly improved, MUSE scores for parents and adolescents did not. This finding suggests that the self-efficacy constructs tested by MUSE are likely unrelated to handling and disposal of opioid prescriptions and, therefore, are not good indicators of behavioral intent or behavioral outcomes.

In addition, parents’ opioid knowledge concept scores did not increase in our study. An interpretation of these results posits that parents already have a high degree of opioid knowledge compared with what our study measures; therefore, there is less content the parents are unfamiliar with in the game and surveys. As the scale used to measure changes in opioid concept scores was created and validated for adolescents, it is likely that the knowledge tested within it is already apparent to parents but not to their children [33,34]. Parents improved in the key concept scores for storage and disposal.

### Limitations

Conclusions of this study face limitations. Although there is diversity in this study’s population, the sample is still largely homogeneous (White and female). Therefore, extrapolating the impact of this intervention to subgroups is not warranted in this study and requires additional research with a large sample that is more diverse. Moreover, owing to the lack of significant improvement in self-efficacy–MUSE and Narcan knowledge, this study can only suggest that future adaptations to the game may provide benefits in these conceptual areas. Cronbach α for several parent concept scores were suboptimal, which may indicate that the survey items used do not accurately measure these concepts at the parent knowledge level. Future iterations of the game will continue to evaluate these concept scores, and game adaptations will be evaluated to find effective means for educating about these topics. Future game adaptations should expose all the players to information about Narcan, not only those who exhibited unsafe behavior.

### Comparison With Previous Studies

Literature has reiterated that adults (including parents of adolescents) are often unaware of safe and responsible practices to store and dispose of opioid medications and regularly retain unused opioids for future use [40]. This lack of awareness and medication retention is owing in part to the counseling practices of providers, who often do not provide substantive information about safe practices regarding storage and disposal [41]. However, previous studies have indicated that when patients are counseled about proper methods for storage and disposal, they are more likely to follow those recommendations [42]. These trends indicate the potential for MedSMAR_x_T: Adventures in PharmaCity to cover a gap in counseling practices and increase safe, sustainable storage and disposal practices in families.

Moreover, this study’s results align with those of previous studies evaluating MedSMAR_x_T: Adventures in PharmaCity wherein adolescents improved across concept scores regardless of whether they had completed the entire game [32]. This effect occurred among parents in this study (except in the case of disposal knowledge) also, providing more comprehensive evidence that even partial play of the game can induce improvement in opioid awareness. In summary, the cumulative results demonstrate that the game can impart crucial knowledge even if players do not play all levels of the game. Participants do not always complete gameplay for a variety of reasons; most commonly, extended gameplay among noncompleters was related to players making unsafe choices and repeating a level. Occasionally, game bugs or control uncertainty may contribute to extended gameplay and noncompletion.

Consistent with the Social Cognitive Theory and Theory of Planned Behavior, increase in perceived knowledge and opioid safety self-efficacy improves behavioral intention and, therefore, the likelihood of participants to practice safe opioid behaviors [43]. Increases in attitude-related concept scores (misuse harm, safe storage, and safe disposal) demonstrate that the game improves attitudes toward safe opioid behaviors, contributing to the increase in favorable behavioral intention. Our results align with those of other studies of Theory of Planned Behavior and health behaviors. Studies have shown that the factors associated with behavioral intention (self-efficacy, attitude, and norms) are correlated with behavioral effects [44].

### Conclusions

This study contributes to a growing body of research demonstrating the intended impacts of serious games in improving health behaviors and, specifically, medication-related outcomes. Serious games are of particular interest to those studying adolescent health owing to the prevalence of gaming. However, future iterations of the game may require changes to the narrative to include a more interactive Narcan-related scenario. That way, adolescents and parents can be more knowledgeable and aware of this life-saving medication and its widespread availability.

This study demonstrates the potential of MedSMAR_x_T: Adventures in PharmaCity to educate youth and their families about the topic of opioid prescriptions: improving self-efficacy and knowledge around safe use, disposal, and handling of these substances. The resulting data elucidate the possibility for serious games to be used in clinical and community pharmacy settings to alleviate time constraints experienced by health care providers, specifically, pharmacists, trying to educate patient populations. The implementation of such educational tools could provide more robust education than what verbal medication counseling can afford in settings such as emergency departments or community pharmacies. The results from this study will support a future randomized controlled trial to rigorously evaluate the effectiveness and implementation of MedSMAR_x_T: Adventures in PharmaCity for clinical, economic, and behavioral outcomes.

## Appendix

Multimedia Appendix 1 Pregame survey.

Multimedia Appendix 2 Postgame survey.

Multimedia Appendix 3 Additional study results.

## Abbreviations

MUSE: Medication Understanding and Use Self-Efficacy
RFCP: Revised Family Communication Patterns

## Data Availability

The data sets generated during and/or analyzed during this study are available from the corresponding author on reasonable request.
