# Supplementary material for: Assessing the Impact of a Serious Game (MedSMARxT: Adventures in PharmaCity) in Improving Opioid Safety Awareness Among Adolescents and Parents: Quantitative Study
Source: JMIR Form Res. 2023 Dec 7;7:e51812. doi: 10.2196/51812 (PMC10739249; doi:10.2196/51812)
Supplement: Multimedia Appendix 1 [file formative_v7i1e51812_app1.pdf]

Demographics

Thank you for consenting to the GAME survey. The first part of your participation will be to complete the following survey.

First, we have some questions about you. These help us understand who took the survey.

What grade are you currently in (2020-21 school year)?

Which one of the following describes how you think of yourself?

- Female
- Male

- Transgender
- Nonbinary
- In another way, please specify:

What is your age?

How many kids under 18 live in your household? *Do not count yourself.*

If you live in more than one home, please use the place where you spend the most time. If you spend equal time in both homes, please select the larger number.

What languages do you speak fluently (select all that apply)?

- English
- Spanish
- Italian
- German
- French

- [ ] Russian
- [ ] Mandarin
- [ ] ______ Other:

Check all of the following that describe your race or ethnicity:

- [ ] American Indian or Alaskan Native
- [ ] Asian
- [ ] Black or African American
- [ ] Hispanic or Latinx
- [ ] Native Hawaiian or Other Pacific Islander
- [ ] White or Caucasian
- [ ] ______ Other, please specify:

Please enter your Zip Code:

______

Pre-game survey

The following questions are about how opioids can affect a person.

Note: Opioids are a class of drugs used to reduce pain.

They include the illegal drug heroin, synthetic opioids such as fentanyl, and pain relievers available legally by prescription, including oxycodone (OxyContin®), hydrocodone (Vicodin®, Percocet®, Roxicodone®), codeine (Tylenol #3), morphine, and tramadol (Ultram®).

We want to know what you think about each of the following.

	Yes	No	Don't Know
Can opioid medications make you dizzy or sleepy even when they are taken as prescribed by your doctor?	○	○	○
If you do not know how much of an opioid medication to take, is it OK to ask your friends?	○	○	○
Can taking too much of an opioid medication cause you to pass out?	○	○	○
Is it safe to drive a car or supervise children after you have taken your prescribed amount of opioid medication?	○	○	○
Is constipation a sign of opioid medication dependence or addiction?	○	○	○

The next questions are about how safe opioids are to take.

Remember: Opioids are a class of drugs used to reduce pain.

They include the illegal drug heroin, synthetic opioids such as fentanyl, and pain relievers available legally by prescription, including oxycodone (OxyContin®), hydrocodone (Vicodin®, Percocet®, Roxicodone®), codeine (Tylenol #3), morphine, and tramadol (Ultram®).

	Yes	No	Don't Know
Can opioid medications cause harm when not used as prescribed by your doctor?	○	○	○
Can extra opioid medications be shared with your friends if they are in pain?	○	○	○
If you take an opioid medication correctly, can there still be side effects?	○	○	○

The next questions are about where to store opioids.

Should prescription opioids be stored...

	Yes	No	Don't know
in the medicine cabinet?	○	○	○
in an unlocked drawer or cabinet?	○	○	○
in a purse or handbag?	○	○	○
in a locked place, such as a lock box, safe, or locked drawer?	○	○	○

The next questions are about what to do with unused opioids.

Should you get rid of unused prescription opioids by...

	Yes	No	Don't know
throwing them in the trash?	○	○	○
dropping them off in a disposal box?	○	○	○
flushing them down the toilet?	○	○	○
putting them in cat litter or coffee grinds?	○	○	○
taking them to a pharmacy, doctor, or hospital?	○	○	○
putting them down the sink/disposal?	○	○	○

How much do you know about...

	None	A little	Some	Quite a bit	g [illegible]
how to use an opioid medication safely?	○	○	○	○	
what counts as misuse of an opioid medication?	○	○	○	○	
the harmful effects of misusing opioids?	○	○	○	○	
please select "a great deal."	○	○	○	○	
how to store opioids safely?	○	○	○	○	
what you should do in situations involving an opioid overdose?	○	○	○	○	
how to dispose of opioids safely?	○	○	○	○	

Now, we'd like to ask more about safe opioid use.

Note: Opioids are a class of drugs used to reduce pain.

They include the illegal drug heroin, synthetic opioids such as fentanyl, and pain relievers available legally by prescription, including oxycodone (OxyContin®), hydrocodone (Vicodin®, Percocet®, Roxicodone®), codeine (Tylenol #3), morphine, and tramadol (Ultram®).

Please tell us how much you agree or disagree with the following statements.

	Strongly disagree	Slightly disagree	Neutral	Slightly agree	Strongly agree
It is easy for me to ask my parent questions about safe opioid use.	○	○	○	○	○
It is easy for me to understand my parent's instructions for using opioids safely.	○	○	○	○	○
It is easy for me to understand instructions on how to safely manage opioids.	○	○	○	○	○
It is easy for me to get all the information I need about safe opioid use.	○	○	○	○	○

How confidant are you that you have the knowledge to...

	Not at all confident	Slightly	Somewhat	Very	Extremely confident
use opioid medication as directed?	○	○	○	○	○

	Not at all confident	Slightly	Somewhat	Very	Extremely confident
know where your medication is at all times?	○	○	○	○	○
store your medication in a locked area?	○	○	○	○	○
dispose of your medication in a dropbox?	○	○	○	○	○
tell a friend no if they ask to share your medication?	○	○	○	○	○
only take medication that was prescribed for you?	○	○	○	○	○
encourage others to use opioids safely?	○	○	○	○	○

How much harm does misuse of opioids do to a person's...

	None	A little	Some	Quite a bit	A gre de
physical health?	○	○	○	○	○
mental health?	○	○	○	○	○
ability to do well in school?	○	○	○	○	○
relationships with their family?	○	○	○	○	○
relationships with their peers or friends?	○	○	○	○	○

Now, we would like to ask you about misuse of prescription opioids.

Is someone misusing opioids if...

	Yes	No	Don't Know
they return their unused opioid medication to the pharmacy when it expires?	○	○	○
they use their prescribed opioid after it expires?	○	○	○
they use someone else's opioid medication?	○	○	○
they use opioids more often than their prescription calls for?	○	○	○
they share their opioid medications with others?	○	○	○
they take their opioid medication for a reason different than what it was prescribed for?	○	○	○

Is the opioid crisis harming teenagers in the U.S.?

- ○ Yes
- ○ No
- ○ Don't know

Have you heard of the drug Naloxone (Narcan®)?

- ○ Yes
- ○ No

Is Naloxone (Narcan®) used to...

	Yes	No	Don't know
reverse only heroin overdoses?	○	○	○
help heroin users detox (the process where addictive toxins leave your body)?	○	○	○
reverse any opioid overdose?	○	○	○

Is it okay to take someone else's opioid medication if you have had the same prescription in the past?

- ○ Yes
- ○ No
- ○ Don't Know

Now, we would like to ask you about what you would do in the future.

How likely are you to do the following in real life?

	Not at all likely	Slightly	Somewhat	Very	Extren like
Use opioid medication as directed?	○	○	○	○	○

	Not at all likely	Slightly	Somewhat	Very	Extrem[illegible] like[illegible]
Know where my medication is at all times?					
Store my medication in a locked area?					
Dispose of my medication in a dropbox?					
Please select "somewhat."					
Share my medication with a friend in need?					
Take medication that was prescribed for someone else?					
Encourage others to use opioids safely?					

After playing an educational video game, would you feel as though your knowledge about opioid medication safety would increase?

- Yes
- No

Behavior

Now, we would like to learn more about how you communicate in your family.

As a reminder we will not share your answers to these

questions with your parent/guardian.

Please use this scale to indicate your agreement with the following statements.

If these statements apply to one or both of your parents/guardians, please answer accordingly.

	Disagree Strongly	Disagree	Neutral	Agree	Agree Strongly
In our family we often talk about topics like politics and religion where some persons disagree with others.	○	○	○	○	○
My parents often say something like “Every member of the family should have some say in family decisions.”	○	○	○	○	○
My parents often ask my opinion when the family is talking about something.	○	○	○	○	○
My parents encourage me to challenge their ideas and beliefs.	○	○	○	○	○

	Disagree Strongly	Disagree	Neutral	Agree	Agree Strongly
My parents often say something like “You should always look at both sides of an issue.”	○	○	○	○	○
I usually tell my parents what I am thinking about things.	○	○	○	○	○
I can tell my parents almost anything.	○	○	○	○	○

	Disagree Strongly	Disagree	Neutral	Agree	Agree Strongly
In our family we often talk about our feelings and emotions.	○	○	○	○	○
My parents and I often have long, relaxed conversations about nothing in particular.	○	○	○	○	○
I really enjoy talking with my parents, even when we disagree.	○	○	○	○	○
My parents encourage me to express my feelings.	○	○	○	○	○
My parents tend to be very open about their emotions.	○	○	○	○	○

	Disagree Strongly	Disagree	Neutral	Agree	Agree Strongly
We often talk as a family about things we have done during the day.	○	○	○	○	○
In our family, we often talk about our plans and hopes for the future.	○	○	○	○	○

	Disagree Strongly	Disagree	Neutral	Agree	Agree Strongly
My parents like to hear my opinion, even when I don't agree with them.	○	○	○	○	○
When anything really important is involved, my parents expect me to obey without question.	○	○	○	○	○
In our home, my parents usually have the last word	○	○	○	○	○
My parents feel that it is important to be the boss.	○	○	○	○	○
My parents sometimes become irritated with my views if they are different from theirs.	○	○	○	○	○
If my parents don't approve of it, they don't want to know about it.	○	○	○	○	○

	Disagree Strongly	Disagree	Neutral	Agree	Agree Strongly
When I am at home, I am expected to obey my parents' rules.	○	○	○	○	○

	Disagree Strongly	Disagree	Neutral	Agree	Agree Strongly
My parents often say things like "You'll know better when you grow up."	○	○	○	○	○
My parents often say things like "My ideas are right and you should not question them."	○	○	○	○	○
My parents often say things like "A child should not argue with adults."	○	○	○	○	○
My parents often say things like "There are some things that just shouldn't be talked about."	○	○	○	○	○
My parents often say things like "You should give in on arguments rather than risk making people mad."	○	○	○	○	○

Mental Health and Opioid Safety Resources

If you are experiencing symptoms of stress, anxiety, depression, or if any of the survey questions elicited feelings of stress, anxiety, or depression and you would like to speak with someone, several supports are available to you. Below is a list of resources you may access.

National resources:

NAMI HELPLINE

800-950-NAMI

info@nami.org

Substance Abuse and Mental Health Services Administration (SAMHSA)

Treatment Referral Helpline

1-800-662-HELP (4357)

If you have questions or concerns about opioid use and safety, please contact your doctor, a school counselor, or refer to www.hhs.gov/opioids.

If you feel you or someone you know is in immediate danger, please call 911.

Thank you for completing the pre-survey!

Your code is: **BLUE**

Please type the code in the WebEx chat, or tell the researcher your code so they know you have finished.

Powered by Qualtrics
