# Supplementary material for: Assessing the Impact of a Serious Game (MedSMARxT: Adventures in PharmaCity) in Improving Opioid Safety Awareness Among Adolescents and Parents: Quantitative Study
Source: JMIR Form Res. 2023 Dec 7;7:e51812. doi: 10.2196/51812 (PMC10739249; doi:10.2196/51812)
Supplement: Multimedia Appendix 2 [file formative_v7i1e51812_app2.pdf]

## Game Evaluation

Which of the following games did you play?

- ○ Eat Right Cafe
- ○ Cancer Fighter
- ○ MedSmart: Adventures in PharmaCity

In two sentences, please describe the game you played in your own words.

The next questions are about how you feel about the game you just played.

| | Strongly disagree | Somewhat disagree | Neither agree nor disagree | Somewhat agree | Strongly agree |
|---|---|---|---|---|---|
| I think that I would like to play this game frequently. | ○ | ○ | ○ | ○ | ○ |
| I found this game too complex. | ○ | ○ | ○ | ○ | ○ |
| I thought this game was easy to use. | ○ | ○ | ○ | ○ | ○ |
| I imagine that most people would learn to play this game very quickly. | ○ | ○ | ○ | ○ | ○ |
| I found this game very awkward to play. | ○ | ○ | ○ | ○ | ○ |
| I felt very confident playing this game. | ○ | ○ | ○ | ○ | ○ |

What aspects about opioid safety did the game best inform you about? (Check all that apply)

- ☐ Safe disposal of opioids
- ☐ How to properly use opioids
- ☐ Safe storage of opioids

- [ ] What to do in the event of an overdose
- [ ] The harmful effects of misusing opioids
- [ ] Other, please specify: ____

What did you like best about this game? (Check all that apply)

- [ ] Graphics
- [ ] Characters
- [ ] The topic
- [ ] The storyline
- [ ] The challenges/obstacles
- [ ] Other, please specify: ____

Please tell us what you thought of the different scenarios.

Which scenario was...

| | Hanging with friends at home | Giving a speech at school | Riding the bus home | Getting rid of medication | None of these |
|---|---|---|---|---|---|
| most fun to play? | ○ | ○ | ○ | ○ | ○ |
| most interesting? | ○ | ○ | ○ | ○ | ○ |
| most difficult to play? | ○ | ○ | ○ | ○ | ○ |
| most informative (e.g. taught you the most)? | ○ | ○ | ○ | ○ | ○ |

What could have been done better in designing this game?

____

Please tell us how much you agree or disagree with the following statements.

| | Strongly disagree | Somewhat disagree | Neutral | Somewhat agree | Strongly agree |
|---|---|---|---|---|---|
| Overall, this is a well-designed game. | ○ | ○ | ○ | ○ | ○ |

| | Strongly disagree | Somewhat disagree | Neutral | Somewhat agree | Strongly agree |
|---|---|---|---|---|---|
| The game is user-friendly. | ○ | ○ | ○ | ○ | ○ |
| The information in this game is reliable. | ○ | ○ | ○ | ○ | ○ |
| The information is personally relevant to me. | ○ | ○ | ○ | ○ | ○ |
| Please select "strongly agree" | ○ | ○ | ○ | ○ | ○ |
| I learned a lot from this game. | ○ | ○ | ○ | ○ | ○ |
| I needed help/technical support while playing this game. | ○ | ○ | ○ | ○ | ○ |
| I would recommend this game to a friend or family member. | ○ | ○ | ○ | ○ | ○ |
| I wanted to continue playing this game. | ○ | ○ | ○ | ○ | ○ |

How experienced are you in electronic games such as gaming on your mobile phone, games for the computer, consoles, etc.?

- ○ NONE: I have no experience in gaming. I do not play games at all.
- ○ BASIC: I have basic gaming experiences.
- ○ MODERATE: I have some gaming experiences.
- ○ INTERMEDIATE: I consider myself experienced.
- ○ EXPERT: I consider myself to be an expert in gaming.

I played the whole MedSMART game: I completed all the levels.

- ○ Yes
- ○ No

If No, what level did you stop at and why?

- ○ Hanging with friends at home
- ○ Monday Morning Bus Ride
- ○ Giving A Speech at School
- ○ Riding the bus home
- ○ Getting rid of medication

○ Please tell us why you stopped here: ______

Please tell us how much you agree or disagree with the following statements.

| | Strongly disagree | Somewhat disagree | Neither agree nor disagree | Somewhat agree | Strongly agree |
|---|---|---|---|---|---|
| When playing this game, my attention was entirely on the game. | ○ | ○ | ○ | ○ | ○ |
| When playing this game, I felt actively involved in the game. | ○ | ○ | ○ | ○ | ○ |
| I could concentrate fully while playing the game. | ○ | ○ | ○ | ○ | ○ |
| The content of the game is educational. | ○ | ○ | ○ | ○ | ○ |
| I can play this game without stopping to think about what buttons to press in order to complete actions within the game. | ○ | ○ | ○ | ○ | ○ |

Where would you be most interested in playing this game? (Check all that apply)

- ☐ In school
- ☐ At home
- ☐ At a doctor's office
- ☐ At a clinic
- ☐ At the hospital
- ☐ At the pharmacy
- ☐ Other, please specify: ______

For this section, please move the slider for each word to the corresponding number to indicate your rating.

On a scale of 1 -10 (**1** being ***not at all*** and **10** being ***extremely***) to me, this game is...

| | 0 | 1 | 2 | 3 | 4 | 5 | 6 | 7 | 8 | 9 | 10 |
|---|---|---|---|---|---|---|---|---|---|---|---|
| **Important** | | | | | | | | | | | |
| **Boring** | | | | | | | | | | | |
| **Realistic** | | | | | | | | | | | |
| **Exciting** | | | | | | | | | | | |
| **Means nothing** | | | | | | | | | | | |
| **Fascinating** | | | | | | | | | | | |
| **Involving** | | | | | | | | | | | |
| **Not needed** | | | | | | | | | | | |

## Prior Experience

Have you ever been prescribed an opioid by a doctor before?

***Note:*** **Opioids are a class of drugs used to reduce pain.**

They include the illegal drug heroin, synthetic opioids such as fentanyl, and pain relievers available legally by prescription, including oxycodone (OxyContin®), hydrocodone (Vicodin®, Percocet®, Roxicodone®), codeine (Tylenol #3), morphine, and tramadol (Ultram®).

| Yes | Unsure | No | Prefer not to answer |
|---|---|---|---|
| ○ | ○ | ○ | ○ |

Please list the opioids you have been prescribed.

*Here is a list of commonly prescribed opioids:*
*hydrocodone (Vicodin®) , oxycodone (OxyContin®, Percocet®), oxymorphone (Opana®), morphine (Kadian®, Avinza®), codeine (Tyenol #3), fentanyl and tramadol (Ultram®)*

Please list medications that you have been prescribed that you think may be opioids.

## Post-game opioid safety

The following questions are about how opioids can affect a person.

***Note: Opioids are a class of drugs used to reduce pain.***

*They include the illegal drug heroin, synthetic opioids such as fentanyl, and pain relievers available legally by prescription, including oxycodone (OxyContin®), hydrocodone (Vicodin®, Percocet®, Roxicodone®), codeine (Tylenol #3), morphine, and tramadol (Ultram®).*

We want to know what you think about each of the following.

| | Yes | No | Don't Know |
|---|---|---|---|
| Can opioid medications make you dizzy or sleepy even when they are taken as prescribed by your doctor? | O | O | O |
| If you do not know how much of an opioid medication to take, is it OK to ask your friends? | O | O | O |
| Can taking too much of an opioid medication cause you to pass out? | O | O | O |
| Is it safe to drive a car or supervise children after you have taken your prescribed amount of opioid medication? | O | O | O |
| Is constipation a sign of opioid medication dependence or addiction? | O | O | O |

The next questions are about how safe opioids are to take.

***Remember:* Opioids are a class of drugs used to reduce pain.**

*They include the illegal drug heroin, synthetic opioids such as fentanyl, and pain relievers available legally by prescription, including oxycodone (OxyContin®), hydrocodone (Vicodin®, Percocet®, Roxicodone®), codeine (Tylenol #3), morphine, and tramadol (Ultram®).*

| | Yes | No | Don't Know |
|---|---|---|---|
| Can opioid medications cause harm when not used as prescribed by your doctor? | O | O | O |
| Can extra opioid medications be shared with your friends if they are in pain? | O | O | O |

| | Yes | No | Don't Know |
|---|---|---|---|
| If you take an opioid medication correctly, can there still be side effects? | ○ | ○ | ○ |

The next questions are about where to store opioids.

Should prescription opioids be stored...

| | Yes | No | Don't know |
|---|---|---|---|
| in the medicine cabinet? | ○ | ○ | ○ |
| in an unlocked drawer or cabinet? | ○ | ○ | ○ |
| in a purse or handbag? | ○ | ○ | ○ |
| in a locked place, such as a lock box, safe, or locked drawer? | ○ | ○ | ○ |

The next questions are about what to do with unused opioids.

Should you get rid of unused prescription opioids by...

| | Yes | No | Don't know |
|---|---|---|---|
| throwing them in the trash? | ○ | ○ | ○ |
| dropping them off in a disposal box? | ○ | ○ | ○ |
| flushing them down the toilet? | ○ | ○ | ○ |
| putting them in cat litter or coffee grinds? | ○ | ○ | ○ |
| taking them to a pharmacy, doctor, or hospital? | ○ | ○ | ○ |
| putting them down the sink/disposal? | ○ | ○ | ○ |

How much do you know about...

| | None | A little | Some | Quite a bit | A great deal |
|---|---|---|---|---|---|
| how to use an opioid medication safely? | ○ | ○ | ○ | ○ | ○ |
| what counts as misuse of an opioid medication? | ○ | ○ | ○ | ○ | ○ |
| the harmful effects of misusing opioids? | ○ | ○ | ○ | ○ | ○ |
| please select "a great deal." | ○ | ○ | ○ | ○ | ○ |
| how to store opioids safely? | ○ | ○ | ○ | ○ | ○ |

| | None | A little | Some | Quite a bit | A great deal |
|---|---|---|---|---|---|
| what you should do in situations involving an opioid overdose? | ○ | ○ | ○ | ○ | ○ |
| how to dispose of opioids safely? | ○ | ○ | ○ | ○ | ○ |

Now, we'd like to ask more about safe opioid use.

***Remember,* Opioids are a class of drugs used to reduce pain.**

*They include the illegal drug heroin, synthetic opioids such as fentanyl, and pain relievers available legally by prescription, including oxycodone (OxyContin®), hydrocodone (Vicodin®, Percocet®, Roxicodone®), codeine (Tylenol #3), morphine, and tramadol (Ultram®).*

Please tell us how much you agree or disagree with the following statements.

| | Strongly disagree | Slightly disagree | Neutral | Slightly agree | Strongly agree |
|---|---|---|---|---|---|
| It is easy for me to ask my parent questions about safe opioid use. | ○ | ○ | ○ | ○ | ○ |
| It is easy for me to understand my parent's instructions for using opioids safely. | ○ | ○ | ○ | ○ | ○ |
| It is easy for me to understand instructions on how to safely manage opioids. | ○ | ○ | ○ | ○ | ○ |
| It is easy for me to get all the information I need about safe opioid use. | ○ | ○ | ○ | ○ | ○ |

How confidant are you that you have the knowledge to...

| | Not at all confident | Slightly | Somewhat | Very | Extremely confident |
|---|---|---|---|---|---|
| use opioid medication as directed? | ○ | ○ | ○ | ○ | ○ |
| know where your medication is at all times? | ○ | ○ | ○ | ○ | ○ |
| store your medication in a locked area? | ○ | ○ | ○ | ○ | ○ |
| dispose of your medication in a dropbox? | ○ | ○ | ○ | ○ | ○ |
| tell a friend no if they ask to share your medication? | ○ | ○ | ○ | ○ | ○ |

| | Not at all confident | Slightly | Somewhat | Very | Extremely confident |
|---|---|---|---|---|---|
| only take medication that was prescribed for you? | ○ | ○ | ○ | ○ | ○ |
| encourage others to use opioids safely? | ○ | ○ | ○ | ○ | ○ |

How much harm does misuse of opioids do to a person's...

| | None | A little | Some | Quite a bit | A great deal |
|---|---|---|---|---|---|
| physical health? | ○ | ○ | ○ | ○ | ○ |
| mental health? | ○ | ○ | ○ | ○ | ○ |
| ability to do well in school? | ○ | ○ | ○ | ○ | ○ |
| relationships with their family? | ○ | ○ | ○ | ○ | ○ |
| relationships with their peers or friends? | ○ | ○ | ○ | ○ | ○ |

Now, we would like to ask you about misuse of prescription opioids. Is someone misusing opioids if...

| | Yes | No | Don't Know |
|---|---|---|---|
| they return their unused opioid medication to the pharmacy when it expires? | ○ | ○ | ○ |
| they use their prescribed opioid after it expires? | ○ | ○ | ○ |
| they use someone else's opioid medication? | ○ | ○ | ○ |
| they use opioids more often than their prescription calls for? | ○ | ○ | ○ |
| they share their opioid medications with others? | ○ | ○ | ○ |
| they take their opioid medication for a reason different than what it was prescribed for? | ○ | ○ | ○ |

Is the opioid crisis harming teenagers in the U.S.?

- ○ Yes
- ○ No
- ○ Don't know

Have you heard of the drug naloxone (Narcan®)?

- ◯ Yes
- ◯ No

Is Naloxone (Narcan®) used to...

| | Yes | No | Don't know |
|---|---|---|---|
| a. reverse only heroin overdoses? | ◯ | ◯ | ◯ |
| b. help heroin users detox (the process where addictive toxins leave your body)? | ◯ | ◯ | ◯ |
| c. reverse any opioid overdose? | ◯ | ◯ | ◯ |

Is it okay to take someone else's opioid medication if you have had the same prescription in the past?

- ◯ Yes
- ◯ No
- ◯ Don't Know

Now, we would like to ask you about what you would do in the future.

How likely are you to do the following in real life?

| | Not at all likely | Slightly | Somewhat | Very | Extremely likely |
|---|---|---|---|---|---|
| Use opioid medication as directed? | ◯ | ◯ | ◯ | ◯ | ◯ |
| Know where my medication is at all times? | ◯ | ◯ | ◯ | ◯ | ◯ |
| Store my medication in a locked area? | ◯ | ◯ | ◯ | ◯ | ◯ |
| Dispose of my medication in a dropbox? | ◯ | ◯ | ◯ | ◯ | ◯ |
| Share my medication with a friend in need? | ◯ | ◯ | ◯ | ◯ | ◯ |
| Please select "somewhat." | ◯ | ◯ | ◯ | ◯ | ◯ |
| Take medication that was prescribed for someone else? | ◯ | ◯ | ◯ | ◯ | ◯ |
| Encourage others to use opioids safely? | ◯ | ◯ | ◯ | ◯ | ◯ |

## STAI

# Mental Health and Opioid Safety Resources

If you are experiencing symptoms of stress, anxiety, depression, or if any of the survey questions elicited feelings of stress, anxiety, or depression and you would like to speak with someone, several supports are available to you. Below is a list of resources you may access.

**National resources:**
**NAMI HELPLINE**
800-950-NAMI
info@nami.org

**Substance Abuse and Mental Health Services Administration (SAMHSA)**
Treatment Referral Helpline
1-800-662-HELP (4357)

If you have questions or concerns about opioid use and safety, please contact your doctor, a school counselor, or refer to www.hhs.gov/opioids.

If you feel you or someone you know is in immediate danger, please call 911.

Thank you for completing the post-survey!
Your code is: GREEN
Please type the code in the WebEx chat, or tell the researcher your code so they know you have finished.

Powered by Qualtrics
