# Supplementary material for: Assessing the Impact of a Serious Game (MedSMARxT: Adventures in PharmaCity) in Improving Opioid Safety Awareness Among Adolescents and Parents: Quantitative Study
Source: JMIR Form Res. 2023 Dec 7;7:e51812. doi: 10.2196/51812 (PMC10739249; doi:10.2196/51812)
Supplement: Multimedia Appendix 3 [file formative_v7i1e51812_app3.docx]

**Supplementary File 3**

Of note, some differences were recorded relating to gender, race, and ethnicity in child participants. Female participants had greater improvement than males for Opioid Knowledge (female mean (sd) = 0.14 (0.16), male = 0.01 (0.21), p = 0.006). Self-efficacy: Opioid Safety for participants who did not identify as White or were Hispanic had greater improvements than White participants (non-White mean (sd) = 0.52 (0.56), White = 0.23 (0.61), p = 0.035). Self-efficacy: Opioid Safety for participants who were Black or African American (mean (sd) = 0.78 (0.43)) had greater improvements than White participants (mean (sd) = 0.23 (0.61), p = 0.006) and Hispanic or Latinx participants (mean (sd) = 0.20 (0.52), p = 0.020). Misuse Harm for participants who were Hispanic or Latinx (mean (sd) = 0.22 (0.23)) had greater improvements than White participants (mean (sd) = -0.17 (0.78), p = 0.0363) and participants of other races (mean (sd) = -0.14 (0.15), p = 0.0048).

**Table S1.** Child Results: Gender, Race and Ethnicity Differences

| **Characteristics** | | **Self-efficacy: MUSE^a^** | **Self-efficacy: opioid safety** | **Perceived knowledge** | **Misuse harm** | **Behavioral intent** | **Safe storage** | **Safe disposal** | **Opioid knowledge** | **Narcan knowledge** | **Misuse behavior** |
| --- | --- | --- | --- | --- | --- | --- | --- | --- | --- | --- | --- |
| By Gender | Female, mean (SD) | -0.18 (0.75) | 0.40 (0.75) | 0.88 (1.49) | -0.15 (0.96) | 0.41 (0.40) | 0.08 (0.23) | 0.08 (0.22) | **0.14 (0.16)** | 0.00 (0.00) | 0.08 (0.19) |
|  | Male, mean (SD) | 0.21 (0.88) | 0.31 (0.52) | 1.14 (0.72) | 0.01 (0.38) | 0.17 (0.37) | 0.15 (0.30) | 0.09 (0.29) | **0.01 (0.21)** | 0.19 (0.38) | 0.03 (0.11) |
|  | Kruskal-Wallis p-value | 0.104 | 0.277 | 0.85 | 0.838 | 0.076 | 0.342 | 0.895 | **0.006** | 0.423 | 0.318 |
| By Race (Grouping 1) | White or Caucasian, mean (SD) | -0.07 (0.72) | **0.23 (0.61)** | 1.10 (1.13) | **-0.17 (0.78)** | 0.32 (0.38) | 0.14 (0.32) | 0.14 (0.32) | 0.10 (0.21) | -0.08 (0.24) | 0.01 (0.07) |
|  | Black or African American, mean (SD) | 0.06 (0.39) | **0.78 (0.43)** | 1.04 (1.17) | **0.18 (0.51)** | 0.02 (0.48) | 0.11 (0.18) | -0.00 (0.20) | 0.00 (0.23) | 0.33 (NA) | 0.07 (0.12) |
|  | Hispanic or Latinx, mean (SD) | 0.72 (1.39) | **0.20 (0.52)** | 1.21 (0.92) | **0.22 (0.23)** | 0.30 (0.42) | 0.09 (0.13) | 0.06 (0.20) | 0.05 (0.09) | 0.50 (0.71) | 0.08 (0.18) |
|  | Other or missing, mean (SD) | 0.11 (0.69) | **0.55 (0.64)** | 0.95 (0.62) | **-0.14 (0.15)** | 0.27 (0.19) | 0.07 (0.12) | 0.05 (0.08) | 0.14 (0.17) | 0.00 (0.00) | 0.14 (0.31) |
|  | Kruskal-Wallis p-value | 0.334 | **0.036** | 0.871 | **0.04** | 0.447 | 0.911 | 0.979 | 0.564 | 0.1 | 0.317 |
| By Race (Grouping 2)^b^ | White or Caucasian, mean (SD) | -0.07 (0.72) | **0.23 (0.61)** | 1.10 (1.13) | -0.17 (0.78) | 0.32 (0.38) | 0.14 (0.32) | 0.14 (0.32) | 0.10 (0.21) | -0.08 (0.24) | 0.01 (0.07) |
|  | Not white, mean (SD) | 0.29 (0.93) | **0.52 (0.56)** | 1.07 (0.92) | 0.10 (0.37) | 0.18 (0.40) | 0.09 (0.14) | 0.03 (0.17) | 0.06 (0.18) | 0.27 (0.43) | 0.10 (0.20) |
|  | Kruskal-Wallis p-value | 0.274 | **0.035** | 0.632 | 0.525 | 0.7 | 0.518 | 0.801 | 0.435 | 0.06 | 0.061 |
| By School | High school, mean (SD) | -0.08 (0.65) | 0.35 (0.68) | 1.06 (1.24) | -0.10 (0.84) | 0.26 (0.41) | 0.09 (0.23) | 0.07 (0.29) | **0.13 (0.20)** | -0.11 (0.27) | 0.02 (0.09) |
|  | Middle school, mean (SD) | 0.20 (0.93) | 0.35 (0.54) | 1.10 (0.87) | -0.02 (0.46) | 0.27 (0.38) | 0.14 (0.29) | 0.12 (0.26) | **0.04 (0.19)** | 0.19 (0.38) | 0.07 (0.18) |
|  | Kruskal-Wallis p-value | 0.507 | 0.765 | 0.702 | 0.894 | 0.454 | 0.619 | 0.299 | **0.028** | 0.101 | 0.421 |
| By Grade | Correlation coefficient | -0.06 | 0.02 | 0.01 | -0.01 | 0.04 | -0.05 | -0.02 | 0.21 | -0.39 | -0.11 |
|  | 95% CI | -0.30 to 0.20 | -0.24 to 0.27 | -0.24 to 0.27 | -0.26 to 0.25 | -0.22 to 0.29 | -0.30 to 0.21 | -0.27 to 0.24 | -0.05 to 0.44 | -0.78 to 0.20 | -0.36 to 0.14 |
|  | Kendall's correlation p-value | 0.843 | 0.876 | 0.831 | 0.805 | 0.163 | 0.566 | 0.372 | 0.071 | 0.09 | 0.588 |
| By Age | Correlation coefficient | 0.03 | 0.01 | 0.03 | 0 | -0.05 | -0.05 | -0.02 | 0.15 | -0.04 | -0.14 |
|  | 95% CI | -0.22 to 0.29 | -0.25 to 0.26 | -0.23 to 0.28 | -0.25 to 0.26 | -0.31 to 0.21 | -0.30 to 0.21 | -0.28 to 0.23 | -0.11 to 0.39 | -0.58 to 0.52 | -0.38 to 0.12 |
|  | Kendall's correlation p-value | 0.707 | 0.845 | 0.718 | 0.826 | 0.374 | 0.537 | 0.24 | 0.233 | 0.548 | 0.713 |
| By parent’s employment | Full time, mean (sd) | 0.04 (0.55) | 0.40 (0.55) | **1.42 (0.68)** | 0.04 (0.38) | 0.28 (0.26) | 0.16 (0.28) | 0.09 (0.22) | 0.10 (0.16) | -0.17 (0.33) | 0.01 (0.10) |
|  | Part time, unemployed, retired, and other, mean (sd) | 0.10 (1.05) | 0.26 (0.64) | **0.76 (1.25)** | -0.10 (0.82) | 0.23 (0.48) | 0.10 (0.26) | 0.08 (0.28) | 0.05 (0.22) | 0.12 (0.35) | 0.05 (0.10) |
|  | Kruskal-Wallis p-value | 0.94 | 0.58 | **0.017** | 0.452 | 0.571 | 0.175 | 0.648 | 0.607 | 0.151 | 0.135 |
| By income | $100,001 - $250,000, mean (sd) | -0.04 (0.62) | 0.38 (0.54) | **1.38 (0.64)** | 0.04 (0.42) | 0.25 (0.41) | 0.13 (0.28) | 0.08 (0.30) | 0.08 (0.19) | -0.22 (0.38) | 0.03 (0.10) |
|  | $50,001 - $100,000, mean (sd) | 0.39 (1.01) | 0.33 (0.53) | **0.90 (0.96)** | 0.08 (0.30) | 0.31 (0.34) | 0.13 (0.28) | 0.07 (0.16) | 0.07 (0.13) | 0.20 (0.45) | 0.04 (0.12) |
|  | $25,001 - $50,000, mean (sd) | -0.41 (0.95) | 0.05 (0.88) | **0.25 (1.80)** | -0.45 (1.44) | 0.18 (0.46) | 0.06 (0.18) | 0.12 (0.29) | 0.09 (0.34) | 0.00 (0.00) | 0.00 (0.00) |
|  | Under $25,000, mean (sd) | 0.38 (0.53) | 0.57 (0.81) | **2.00 (1.41)** | -0.30 (0.14) | 0.07 (0.30) | 0.25 (0.35) | 0.08 (0.12) | -0.06 (0.09) | 0.00 (NA) | 0.08 (0.12) |
|  | Jonckheere-Terpstra p-value | 0.328 | 0.845 | **0.048** | 0.522 | 0.426 | 0.786 | 0.456 | 0.994 | 0.61 | 0.994 |
| By parent’s education | Associates or trade school, mean (sd) | 0.04 (1.33) | 0.39 (1.15) | 0.45 (2.43) | -0.69 (1.48) | 0.24 (0.37) | 0.14 (0.20) | 0.05 (0.08) | 0.12 (0.18) | 0.00 (0.00) | 0.10 (0.13) |
|  | Bachelor's degree, mean (sd) | -0.05 (0.46) | 0.34 (0.44) | 1.11 (0.65) | 0.16 (0.39) | 0.17 (0.45) | 0.06 (0.25) | 0.10 (0.33) | 0.05 (0.25) | 0.00 (0.00) | 0.03 (0.06) |
|  | High school, mean (sd) | 0.92 (2.74) | 0.52 (0.54) | 0.83 (1.15) | 0.13 (0.23) | 0.81 (0.50) | 0.25 (0.43) | 0.17 (0.29) | 0.04 (0.14) | 1.00 (NA) | 0.11 (0.19) |
|  | Master's or PhD, mean (sd) | 0.10 (0.55) | 0.29 (0.54) | 1.30 (0.71) | -0.01 (0.36) | 0.26 (0.30) | 0.17 (0.29) | 0.07 (0.23) | 0.06 (0.16) | -0.13 (0.30) | 0.02 (0.10) |
|  | Jonckheere-Terpstra p-value | 0.884 | 0.272 | 0.288 | 0.81 | 0.758 | 0.6 | 0.561 | 0.397 | 0.213 | 0.229 |

**Table S2.** Relationship between children’s RFCP and concept scores at baseline and improvement.

| **RFCP** | | **Opioid knowledge** | **Safe Disposal** | **Safe Storage** | **Self-efficacy: Learning Objectives** | **Self-efficacy: MUSE** |
| --- | --- | --- | --- | --- | --- | --- |
| **Baseline** | |  |  |  |  |  |
| Conformity | Correlation coefficient | 0.14 | 0.01 | 0.17 | 0.07 | -0.13 |
|  | 95% CI | -0.12 to 0.39 | -0.25 to 0.27 | -0.09 to 0.41 | -0.19 to 0.32 | -0.37 to 0.13 |
|  | Pearson's correlation p-value | 0.274 | 0.934 | 0.208 | 0.624 | 0.334 |
| Conversation | Correlation coefficient | 0.22 | 0.16 | 0.11 | 0.22 | 0.26 |
|  | 95% CI | -0.04 to 0.45 | -0.10 to 0.41 | -0.15 to 0.36 | -0.04 to 0.45 | -0.00 to 0.48 |
|  | Pearson's correlation p-value | 0.103 | 0.219 | 0.414 | 0.095 | 0.053 |
| **Pre/post change** | |  |  |  |  |  |
| Conformity | Correlation coefficient | -0.1 | 0.01 | -0.15 | 0.05 | 0.13 |
|  | 95% CI | -0.35 to 0.16 | -0.24 to 0.27 | -0.39 to 0.11 | -0.21 to 0.30 | -0.13 to 0.38 |
|  | Pearson's correlation p-value | 0.437 | 0.913 | 0.246 | 0.69 | 0.313 |
| Conversation | Correlation coefficient | **-0.28** | -0.18 | -0.04 | -0.03 | 0.16 |
|  | 95% CI | **-0.50 to -0.03** | -0.42 to 0.08 | -0.30 to 0.22 | -0.29 to 0.23 | -0.11 to 0.40 |
|  | Pearson's correlation p-value | **0.031** | 0.183 | 0.738 | 0.798 | 0.241 |

**Parent Results**

Gender, racial and ethnic differences were also reported in parents. Male participants had greater improvement than females for safe disposal (male mean (sd) = 0.20 (0.27), female = 0.06 (0.14), p = 0.047) and self-efficacy: Opioid Safety (male mean (sd) = 0.69 (0.47), female = 0.24 (0.41), p = 0.022). Behavioral intent for participants who identified as White had greater improvements than participants who did not identify as White or were Hispanic (White mean (sd) = 0.30 (0.31), non-White = 0.09 (0.30), p = 0.037). Safe disposal for participants who were Hispanic or Latinx (mean (sd) = 0.21 (0.27)) had greater improvements than White participants (mean (sd) = 0.05 (0.13), p = 0.025) and Black or African American participants (mean (sd) = -0.03 (0.07), p = 0.028).

**Table S3.** Parent Results: Gender, Race and Ethnicity Differences

| **Characteristics** | | **Self-efficacy: MUSE^a^** | **Self-efficacy: opioid safety** | **Perceived knowledge** | **Misuse harm** | **Behavioral intent** | **Safe storage** | **Safe disposal** | **Opioid knowledge** | **Narcan knowledge** | **Misuse behavior** |
| --- | --- | --- | --- | --- | --- | --- | --- | --- | --- | --- | --- |
| By gender | Female, mean (sd) | 0.09 (0.56) | **0.24 (0.41)** | 0.55 (0.56) | 0.07 (0.38) | 0.24 (0.32) | 0.03 (0.11) | **0.06 (0.14)** | 0.01 (0.08) | -0.00 (0.21) | 0.04 (0.10) |
|  | Male, mean (sd) | 0.10 (0.22) | **0.69 (0.47)** | 0.73 (0.55) | 0.40 (0.69) | 0.49 (0.24) | 0.05 (0.11) | **0.20 (0.27)** | 0.00 (0.00) | 0.00 (0.00) | 0.03 (0.07) |
|  | Kruskal-Wallis p-value | 0.749 | **0.022** | 0.333 | 0.206 | 0.059 | 0.403 | **0.047** | 0.808 | 0.868 | 0.924 |
| By race (grouping 1) | White or Caucasian, mean (sd) | 0.06 (0.53) | 0.30 (0.45) | 0.54 (0.51) | 0.05 (0.34) | 0.30 (0.31) | 0.03 (0.11) | **0.05 (0.13)** | 0.00 (0.06) | -0.00 (0.21) | 0.04 (0.09) |
|  | Black or African American, mean (sd) | 0.42 (0.49) | 0.14 (0.20) | 0.81 (0.61) | 0.17 (0.41) | 0.02 (0.11) | 0.04 (0.10) | **-0.03 (0.07)** | 0.06 (0.10) | 0.11 (0.19) | 0.03 (0.07) |
|  | Hispanic or Latinx, mean (sd) | 0.00 (0.65) | 0.20 (0.45) | 0.40 (0.60) | 0.20 (0.68) | 0.08 (0.40) | 0.07 (0.19) | **0.21 (0.27)** | 0.05 (0.10) | -0.07 (0.15) | 0.10 (0.19) |
|  | Other or missing, mean (sd) | 0.25 (0.35) | 0.21 (0.30) | 1.00 (1.41) | 0.80 (0.57) | 0.29 (0.40) | 0.00 (0.00) | **0.17 (0.24)** | -0.12 (0.18) | 0.00 (NA) | 0.00 (0.00) |
|  | Kruskal-Wallis p-value | 0.263 | 0.957 | 0.604 | 0.092 | 0.132 | 0.805 | **0.021** | 0.05 | 0.589 | 0.824 |
| By race (grouping 2) | White or Caucasian, mean (sd) | 0.06 (0.53) | 0.30 (0.45) | 0.54 (0.51) | 0.05 (0.34) | **0.30 (0.31)** | 0.03 (0.11) | 0.05 (0.13) | 0.00 (0.06) | -0.00 (0.21) | 0.04 (0.09) |
|  | Not white, mean (sd) | 0.20 (0.56) | 0.18 (0.33) | 0.64 (0.70) | 0.27 (0.57) | **0.09 (0.30)** | 0.05 (0.14) | 0.11 (0.22) | 0.03 (0.12) | 0.00 (0.17) | 0.06 (0.14) |
|  | Kruskal-Wallis p-value | 0.196 | 0.63 | 0.777 | 0.196 | **0.037** | 0.489 | 0.459 | 0.139 | 0.828 | 0.992 |
| By employment | Full time, mean (sd) | 0.09 (0.62) | 0.19 (0.33) | 0.61 (0.51) | 0.09 (0.46) | 0.27 (0.31) | **0.00 (0.00)** | 0.05 (0.13) | 0.00 (0.04) | 0.04 (0.17) | 0.03 (0.07) |
|  | Part time, unemployed, retired, and other, mean (sd) | 0.09 (0.46) | 0.36 (0.49) | 0.51 (0.60) | 0.10 (0.36) | 0.24 (0.33) | **0.06 (0.15)** | 0.09 (0.17) | 0.01 (0.10) | -0.03 (0.22) | 0.06 (0.13) |
|  | Kruskal-Wallis p-value | 0.681 | 0.211 | 0.361 | 0.589 | 0.454 | **0.014** | 0.201 | 0.741 | 0.262 | 0.518 |
| By income | $100,001 - $250,000, mean (sd) | 0.00 (0.33) | 0.31 (0.41) | 0.56 (0.47) | 0.07 (0.44) | 0.30 (0.33) | 0.02 (0.06) | 0.08 (0.16) | 0.01 (0.05) | 0.01 (0.19) | 0.04 (0.08) |
|  | $25,001 - $50,000, mean (sd) | 0.12 (0.64) | 0.05 (0.13) | 0.56 (0.56) | 0.10 (0.21) | 0.14 (0.22) | 0.12 (0.23) | 0.15 (0.27) | 0.03 (0.11) | -0.20 (0.30) | 0.06 (0.20) |
|  | $250,001 - $500,000, mean (sd) | 0.00 (0.00) | 0.00 (0.00) | -0.25 (0.35) | 0.00 (0.00) | 0.14 (0.20) | 0.00 (0.00) | 0.08 (0.12) | 0.00 (0.00) | 0.00 (0.00) | 0.00 (0.00) |
|  | $50,001 - $100,000, mean (sd) | 0.22 (0.75) | 0.29 (0.49) | 0.67 (0.63) | 0.12 (0.44) | 0.25 (0.36) | 0.03 (0.11) | 0.03 (0.10) | 0.00 (0.10) | 0.03 (0.18) | 0.03 (0.06) |
|  | Under $25,000, mean (sd) | 0.00 (0.00) | 0.79 (0.30) | 0.25 (0.82) | 0.30 (0.42) | 0.07 (0.10) | 0.00 (0.00) | 0.00 (0.00) | 0.00 (0.18) | 0.00 (0.00) | 0.25 (0.12) |
|  | Jonckheere-Terpstra p-value | 0.489 | 0.657 | 0.56 | 0.324 | 0.144 | 0.533 | 0.464 | 0.991 | 0.488 | 0.587 |
| By age | Correlation coefficient | -0.19 | -0.08 | -0.16 | -0.04 | -0.11 | -0.24 | -0.14 | -0.22 | -0.14 | -0.32 |
|  | 95% CI | -0.41 to 0.05 | -0.31 to 0.16 | -0.38 to 0.08 | -0.27 to 0.20 | -0.34 to 0.13 | -0.45 to 0.00 | -0.36 to 0.10 | -0.43 to 0.02 | -0.39 to 0.12 | -0.52 to -0.08 |
|  | Kendall's correlation p-value | 0.108 | 0.661 | 0.206 | 0.332 | 0.605 | 0.573 | 0.353 | 0.267 | 0.319 | 0.092 |
| By education | Associates or trade school, mean (sd) | 0.19 (0.53) | 0.37 (0.48) | 0.52 (0.55) | -0.05 (0.44) | **0.04 (0.30)** | 0.03 (0.09) | 0.02 (0.06) | 0.02 (0.08) | -0.00 (0.18) | 0.06 (0.12) |
|  | Bachelor's degree, mean (sd) | 0.08 (0.66) | 0.25 (0.33) | 0.51 (0.60) | 0.11 (0.48) | **0.23 (0.33)** | 0.05 (0.14) | 0.11 (0.21) | 0.00 (0.09) | 0.05 (0.22) | 0.03 (0.09) |
|  | High school, mean (sd) | 0.00 (0.00) | 0.43 (0.62) | 0.22 (0.54) | 0.00 (0.00) | **0.29 (0.49)** | 0.08 (0.14) | 0.06 (0.10) | 0.00 (0.00) | -0.11 (0.19) | 0.00 (0.00) |
|  | Master's or PhD, mean (sd) | 0.11 (0.48) | 0.28 (0.50) | 0.66 (0.54) | 0.15 (0.38) | **0.35 (0.29)** | 0.02 (0.09) | 0.05 (0.13) | 0.01 (0.08) | -0.03 (0.19) | 0.07 (0.11) |
|  | Jonckheere-Terpstra p-value | 0.931 | 0.363 | 0.163 | 0.495 | **0.022** | 0.461 | 0.74 | 0.763 | 0.79 | 0.323 |

**Table S4.** Relationship between parents’ RFCP and concept scores at baseline and improvement.

| **RFCP** | | **Opioid knowledge** | **Safe Disposal** | **Safe Storage** | **Self-efficacy: Learning Objectives** | **Self-efficacy: MUSE** |
| --- | --- | --- | --- | --- | --- | --- |
| **Baseline** | |  |  |  |  |  |
| Conformity | Correlation coefficient | -0.01 | -0.04 | -0.14 | -0.13 | -0.09 |
|  | 95% CI | -0.25 to 0.23 | -0.27 to 0.20 | -0.37 to 0.10 | -0.35 to 0.12 | -0.32 to 0.15 |
|  | Pearson's correlation p-value | 0.911 | 0.753 | 0.249 | 0.303 | 0.466 |
| Conversation | Correlation coefficient | **0.28** | 0.21 | 0.18 | 0.13 | **0.26** |
|  | 95% CI | **0.04 to 0.48** | -0.03 to 0.42 | -0.06 to 0.40 | -0.11 to 0.36 | **0.02 to 0.47** |
|  | Pearson's correlation p-value | **0.023** | 0.09 | 0.134 | 0.278 | **0.031** |
| **Pre/post change** | |  |  |  |  |  |
| Conformity | Correlation coefficient | 0.09 | -0.03 | 0.14 | 0.07 | 0.03 |
|  | 95% CI | -0.15 to 0.32 | -0.27 to 0.21 | -0.10 to 0.37 | -0.17 to 0.30 | -0.21 to 0.26 |
|  | Pearson's correlation p-value | 0.477 | 0.808 | 0.249 | 0.581 | 0.82 |
| Conversation | Correlation coefficient | -0.11 | -0.19 | -0.18 | 0.22 | -0.14 |
|  | 95% CI | -0.34 to 0.13 | -0.41 to 0.05 | -0.40 to 0.06 | -0.02 to 0.44 | -0.37 to 0.10 |
|  | Pearson's correlation p-value | 0.357 | 0.12 | 0.134 | 0.068 | 0.245 |
